# Targeted metabolome profiling by dual-probe microdialysis sampling and treatment using *Gardenia jasminoides* for rats with type 2 diabetes

**DOI:** 10.1038/s41598-017-10172-w

**Published:** 2017-08-31

**Authors:** Lu Wang, Zifeng Pi, Shu Liu, Zhiqiang Liu, Fengrui Song

**Affiliations:** 1 National Center of Mass Spectrometry in Changchun, Jilin Province Key Laboratory of Chinese Medicine Chemistry and Mass Spectrometry, Changchun Institute of Applied Chemistry, Chinese Academy of Sciences, Changchun, 130022 China; 2 University of Chinese Academy of Sciences, Beijing, 100039 China

## Abstract

Diabetes causes a variety of end-stage organ complications, including diabetic nephropathy. Metabolomics offers an approach for characterizing biofluid metabolic changes, but studies focusing on diabetic nephropathy are limited due to the loss of tissue-specific metabolic information. A microdialysis application for the sampling of intact endogenous metabolites has been developed, utilizing two probes simultaneously inserted into the kidney tissues and jugular vein of rats with type 2 diabetes. The comprehensive and quantitative analysis of 20 diagnostic biomarkers closely realated to type 2 diabetes and its complications were performed. Results indicated that amino acid and nucleotide levels were lower in diabetic rats, revealing that the metabolic pathways of amino acid, as well as purine and pyrimidine, were disturbed. Targeted metabolomics using mass spectrometry was performed to find potential therapeutic biomarkers and related metabolic pathways of *Gardenia jasminoides* (*G. jasminoides*) for treating diabetes. Results suggested that seven biomarkers in the kidney and five biomarkers in the blood were related to *G. jasminoides*. In addition, the marked perturbations of pathways were regulated after treatment with *G. jasminoides*, including amino acid metabolism and purine metabolism. These biomarkers and metabolic pathways provided new understanding for molecular mechanisms of *G. jasminoides* for treating diabetes and its complications.

## Introduction

Metabolomics strategies can be divided into untargeted and targeted approaches. The emergence of targeted metabolome profiling is an important development in metabolomics. Targeted metabolomics is the measurement of defined groups of metabolites. All or most of the detectable compounds in a given sample were identified and quantified by comparing the sample spectra to a library of reference spectra of pure compounds^1–3^.

For metabolomics strategy, common sample preparation like extraction, concentration with evaporation or freeze-drying, and derivatization steps are essential^4^, but these steps may potentially cause bias and contamination of samples, which may lead to misinterpretation of the final results^5–7^. Therefore, *in vivo* analytical method of biological tissues is one of the ultimate goals for metabolomics to obtain solid information on metabolome alteration. Microdialysis (MD) is a well-established *in vivo* analytical sampling method used over the years for monitoring low-molecular-weight hydrophilic compounds from the interstitial space^8^. MD samples are much cleaner and do not require additional preparation steps that might increase method errors^9^. Furthermore, MD can be used to monitor the metabolic profiles of specific tissues without the need to take tissue biopsies, namely, tissue-targeted metabolomics^9^. Tissue-targeted metabolomics is usually applied for investigating the alterations of metabolic profiles in specific tissues and the effects of which could be lost in the complex metabolic signatures of biofluid, including urine or plasma.

Diabetes mellitus, which is one of the most common endocrine metabolic disorders, can cause microvascular and macrovascular complications. Among type 2 diabetes (T2D) patients, approximately 30% of them evolved a diabetic nephropathy (DN), an end-stage renal disease. DN has been didactically categorized into stages based on the values of urinary albumin excretion rate (UAER): microalbuminuria and macroalbuminuria. The cutoff values adopted by the American Diabetes Association^10^ (timed, 24-h, and spot urine collection) for the diagnosis of microalbuminuria and macroalbuminuria are 30–299 mg/24 h and ≥300 mg/24 h respectively. The basis for the prevention of DN is the treatment of its known risk factors: hypertension, hyperglycemia, smoking, and dyslipidemia^11^. Progress in the development of treatments for DN has been impeded by an incomplete understanding of disease pathogenesis. Aldose reductase (ALR2) has been widely investigated as an enzyme crucially involved in the pathogenesis of chronic complications associated with diabetes. Our previous study^12^ found some components in *G. jasminoides* have high affinity interactions with ALR2 and they were potential aldose reductase inhibitor (ARI). Epalrestat is a noncompetitive and reversible ARI. It reduces the accumulation of intracellular sorbitol which is believed to be the cause of diabetic neuropathy, retinopathy and nephropathy^13^. Epalrestat is the only ARI commercially available. Hence epalrestat was selected as positive control.

Recently, an increasing number of metabolomics studies have been conducted to characterize T2D and its complications^14–19^. Previous studies suggested that metabolic alterations occurred in T2D and its complications. Moreover, a series of metabolites like some amino acids^15, 16^, such as purine, pyrimidine^19^, and sorbital^20^, were identified as diagnostic biomarkers of T2D. Thus, these pivotal metabolites were selected as targeted metabolites in our research. In the present study, we used dual-probe microdialysis combined with liquid chromatography (LC) coupled to a triple quadrupole tandem mass spectrometer to directly detect these important potential biomarkers in the blood and kidney tissue of T2D rats. *G. jasminoides* as traditional Chinese medicines (TCMs) has been used for the treatment of diabetes in clinic. *G. jasminoides* has been reported to improve T2D in rats^21^. However, its therapeutic mechanism is still not very clear. Metabolomics is a powerful approach to explore the multi-target therapeutic mechanism underlying the influence of TCMs. The aim of targeted metabolomics analysis is to determine tissue-specific metabolic profiles and metabolic alterations in response to *G. jasminoides* treatment. In this research, we performed simultaneously qualitative and quantitative analyses of multiple metabolites in the blood and kidney tissues and investigated the mechanism of *G. jasminoides* for treating diabetes and its complications.

## Results

### Method validation

A sensitive LC-MS/MS coupled with dual MD method was validated to be able to simultaneously quantify 20 endogenous compound concentrations from the *in vivo* dialysate. The dynamic range, correlation coefficients, limits of detection (LODs) and limits of quantification (LOQs), and matrix effects for 20 analytes in the analyzed concentration range are shown in Supplementary Table S2. Correlation coefficients, R^2^, were ranged from 0.9920 to 0.9996, revealing good correlations between concentrations and peak areas. The sensitivity of the method was evaluated by determining the LODs and LOQs of the analytes. As listed in Supplementary Table S2, the limit of quantification of this method was from 0.1 ng·mL^−1^ to 10 μg·mL^−1^, which means that the sensitivity is enough for these analytes to be detected in dialysate samples. The LOD range of this method was from 0.075 ng·mL^−1^ to 1.0 ng·mL^−1^. Most  analytes exhibited good repeatability with inter-day and intra-day relative standard deviations (RSDs) lower than 10%, except for a few analytes, which showed an acceptable repeatability with RSDs between 10% and 12%. Moreover, the accuracy were 100% ± 13%, which revealed that the method was valid for *in vivo* dialysate sample analysis. The matrix effects of all analytes ranged from 88.12% to 108.97%, which demonstrated that there was no significant matrix interference in this method (see Supplementary Table S3).

Taken all together, these results illustrate the excellent linearity, precision, and accuracy of this method, as well as the general ability of LC-MRM-MS system to serve in the analysis and quantification of small molecules.

### Biochemical analyses

Supplementary Table S6 shows the biochemical parameters related to renal functions. Levels of renal function markers, urea nitrogen (BUN), serum creatinine (Scr) and urinary albumin excretion rate (UAER) presented significant higher levels (*p* < 0.01) in DM group compared with control group. Value of UAER in DM group suggests that marked DN has been developed on diabetic rats (microalbuminuria stage).

### Endogenous metabolite changes in blood and kidney tissues

Sorbitol, inosine, adenosine, thymidine, cytidine, and cytosine could not be detected or quantified in blood dialysates but could be quantified in kidney tissue dialysates due to the differences in recovery. As a whole, most metabolite concentration levels in kidney tissue dialysates are higher than in blood dialysates.

**Figure 1:**
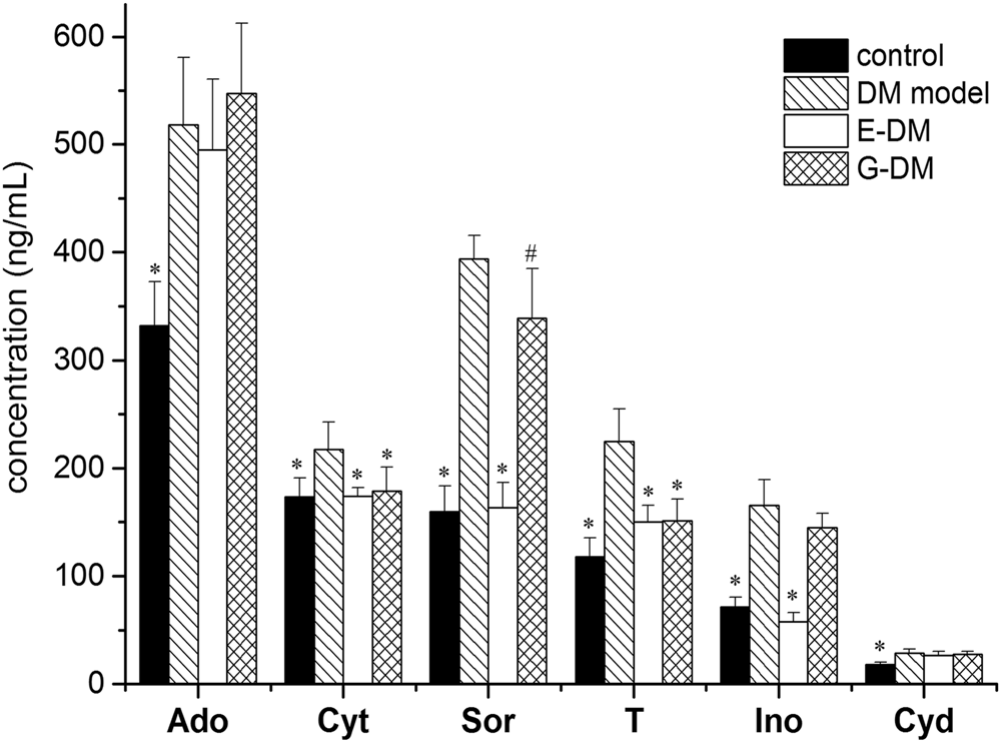
Levels of six analytes only in kidney dialysates among four groups (n = 8 per group). Data presented as mean ± SD. Student’s t test, ^#^ *p* < 0.05, *p < 0.01 vs. DM model group.

In Fig. 1, we can see that the levels of inosine, adenosine, thymidine, cytidine, and cytosine in the group of DM are significantly higher (*p* < 0.01) compared with control group. For the G-DM group, significant lower levels of thymidine and cytosine (*p* < 0.01) compared with DM group indicated the possible mechanism of *G. jasminoides* for treating T2D with DN.

For sorbitol, the concentrations were found to be significantly higher (*p* < 0.01) in DM group than in the control group. Compared with DM group, the level of sorbitol in kidney tissue dialysates was significantly lower (*p* < 0.05) in G-DM treatment group.

**Figure 2:**
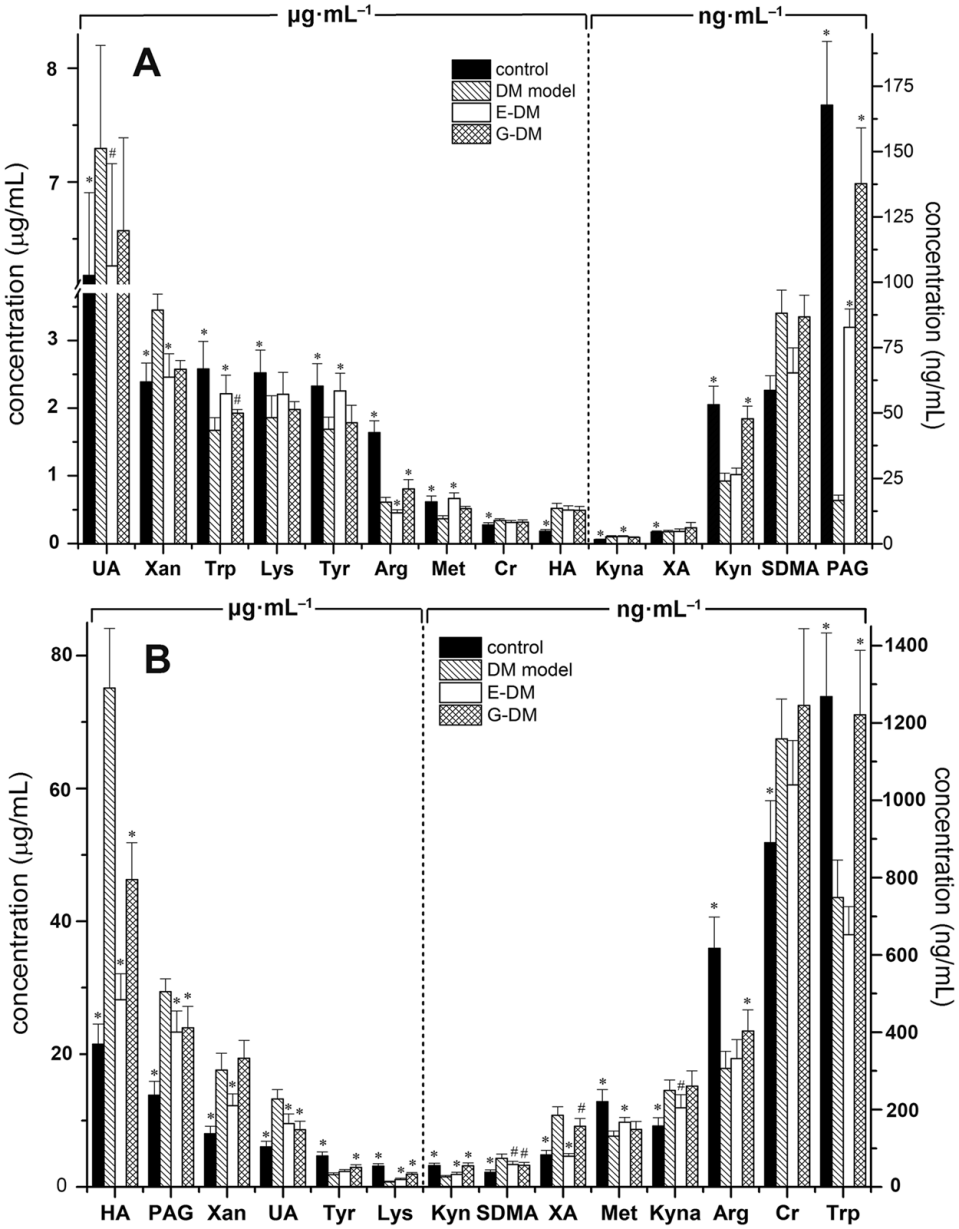
Levels of 14 analytes in blood (**A**) and kidney (**B**) dialysates among four groups (n = 8 per group), respectively. Data presented as mean ± SD. Student’s t test, ^#^ *p* < 0.05, *p < 0.01 vs. DM model group.

The other 14 analytes could be simultaneously quantified in blood and kidney tissue dialysates from rats (see Fig. 2). Compared with the control group, higher level was observed in six analytes, including xanthine, uric acid, SDMA, creatinine, and hippuric acid in the dialysates of blood for diabetic rats. Xanthurenate was significantly higher (*p* < 0.01) in diabetic rats of kidney tissues. However, amino acids and their metabolites, including arginine, tryptophan, kynurenine, kynurenate, phenylacetylglycine, tyrosine, methionine, and lysine levels, were significantly lower (*p* < 0.01) in diabetic rats. In the dialysates of kidney tissues, the ratios of kynurenine to tryptophan in control and DM group were 22.78 and 28.18 respectively, suggesting no significant increase was observed in DM group. However, in the dialysates of blood, the ratios of kynurenine to tryptophan in control and DM group were 48.50 and 69.85 respectively. We noted that the level of phenylacetylglycine in the dialysates of kidney tissues was significantly higher (*p* < 0.01) compared with that in the control group, which had the opposite trend to blood dialysates. All the other analytes in blood and kidney tissues showed similar change trends. Reduction in glomerular filtration rate was reported in diabetic rat^22^, which probably contributes to this phenomenon.

**Figure 3:**
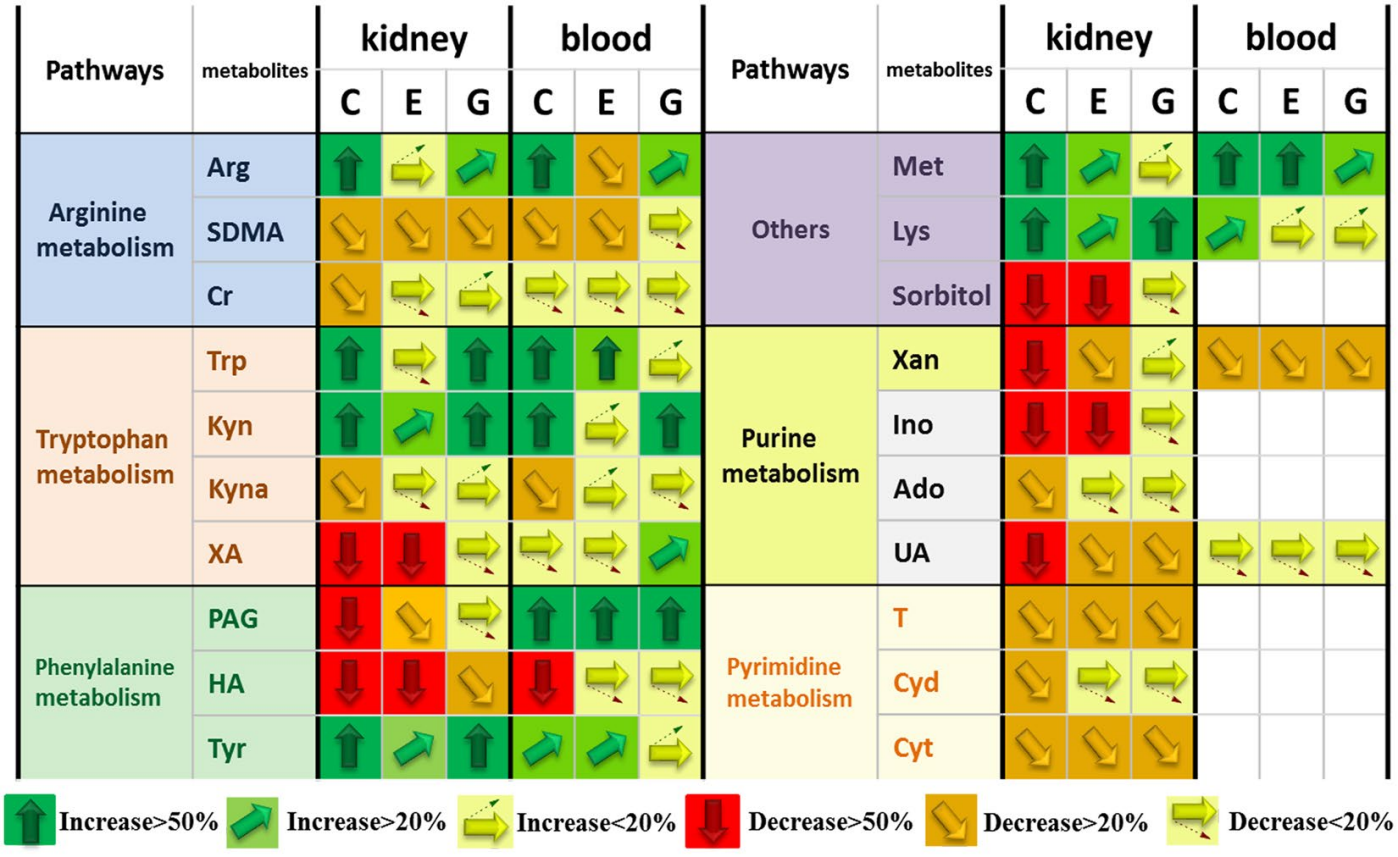
Vector diagram showing the response of the 20 analytes according to their level change. Analytes with vectors are absolutely quantified. Analytes are presented with names, their metabolic pathways (http://www.kegg.com), and their response to intervention for epalrestat (E) and *G. jasminoides* (G) in diabetic rats. C presents control group. The analyte amount in the C, E, and G groups is compared with those in the DM group. An arrow pointing straight up (green) means that the particular analyte has increased >50% vs. DM group. An arrow pointing straight down (red) means that the particular analyte has decreased >50% vs. DM group. White boxes mean that metabolite was not detected or quantified in the samples.

Compared with the DM group, *G. jasminoides* showed a significant treatment effect (*p* < 0.05) in regulating the levels of kynurenate, arginine, tryptophan, and phenylacetylglycine in blood dialysates. By comparison, *G. jasminoides* showed a significant effect (*p* < 0.05) in regulating much more endogenous metabolites in kidney tissue dialysates, including hippuric acid, phenylacetylglycine, uric acid, SDMA, xanthurenate, arginine, tryptophan, kynurenine, kynurenate, phenylacetylglycine, tyrosine, and lysine. As shown in Fig. 3, *G. jasminoides* plays a more important effect in regulating the levels of arginine and tryptophan than epalrestat as a control. Both arginine and tryptophan are pivotal metabolites in amino acid metabolism that associate with T2D^23^. Interestingly, from the quantitative analysis results, we found that *G. jasminoides* exhibited a better therapeutic effect in targeted kidney tissues than blood. Plasma or urine represents the average metabolic status of the organism and cannot give tissue-specific metabolic profiles. MD sampling can offer more tissue-specific metabolic information in kidney tissues, particularly for the research on DN.

### Global metabolome profiling of DM, E-DM, G-DM, and control groups

**Figure 4:**
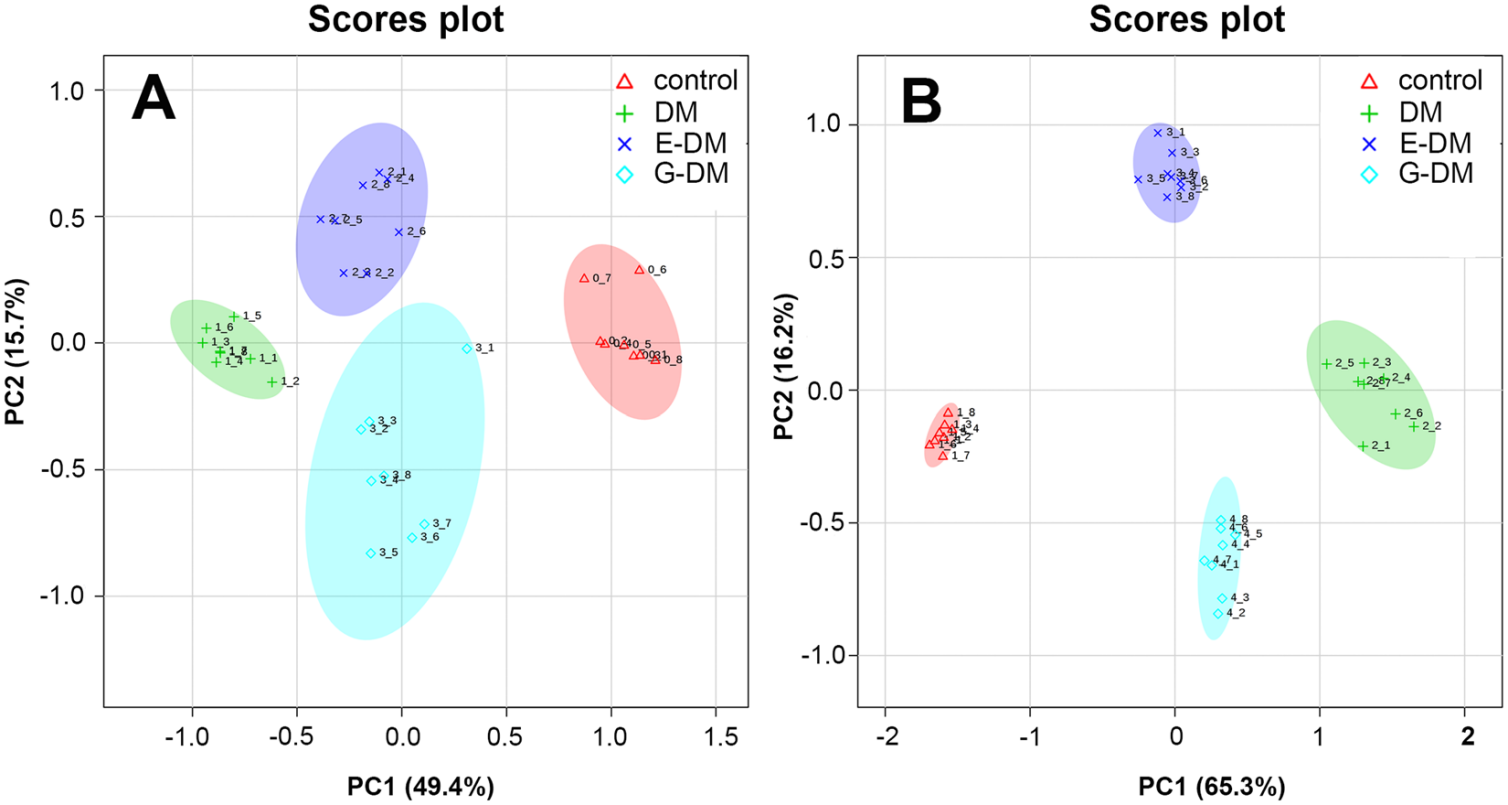
PCA score plots of the control, DM, E-DM, and G-DM groups in blood (**A**) and kidney (**B**) dialysates of the first two principal components. The plot displays PC1 on the x axis and PC2 on the y axis.

To investigate global metabolomic alterations, the concentration data sets obtained from blood and kidney dialysate samples were integrated and analyzed using principal component analysis (PCA). The variance explained by each principal component (PC) is displayed on the X and Y axis (score plot two-dimensional). In Fig. 4(A), PC1 accounts for 49.4% of the variance, PC2 accounts for 15.7% of the variance. In Fig. 4(B), PC1 accounts for 65.3% of the variance, PC2 accounts for 16.2% of the variance. As shown in Fig. 4, a clear separation is observed among the four groups in both blood and kidney dialysates, suggesting that metabolic perturbations were evident in the rats, dependent on pathological condition and treatment intervention.

### Discovery of potential therapeutic biomarkers

**Figure 5:**
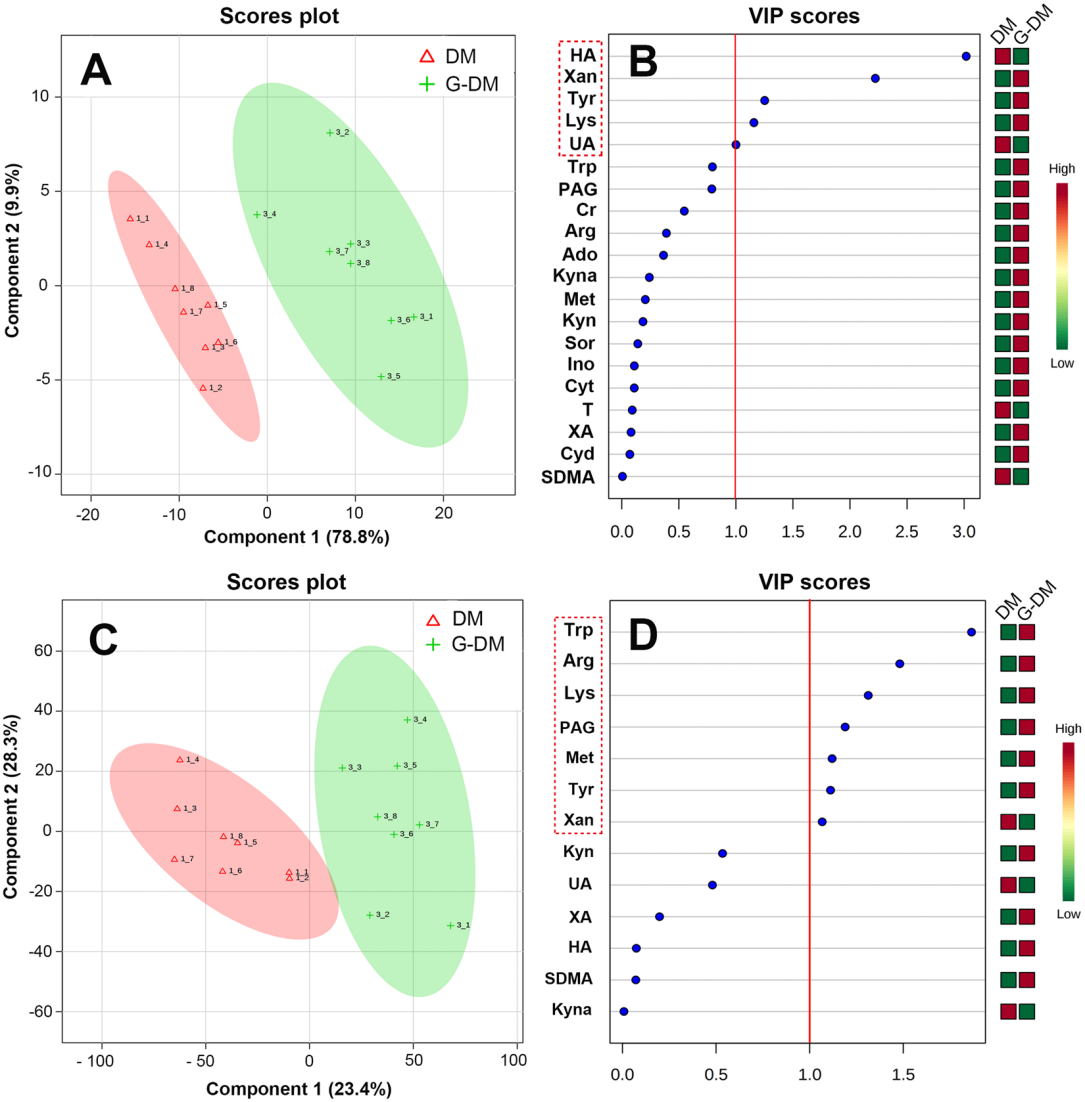
PLS-DA score plots showing a significant separation between G-DM and DM groups in the kidney (**A**) and blood (**C**); VIP scores representing the most contributing metabolites involved in the separation between G-DM and DM groups in the kidney (**B**) and blood (**D**).

**Figure 6:**
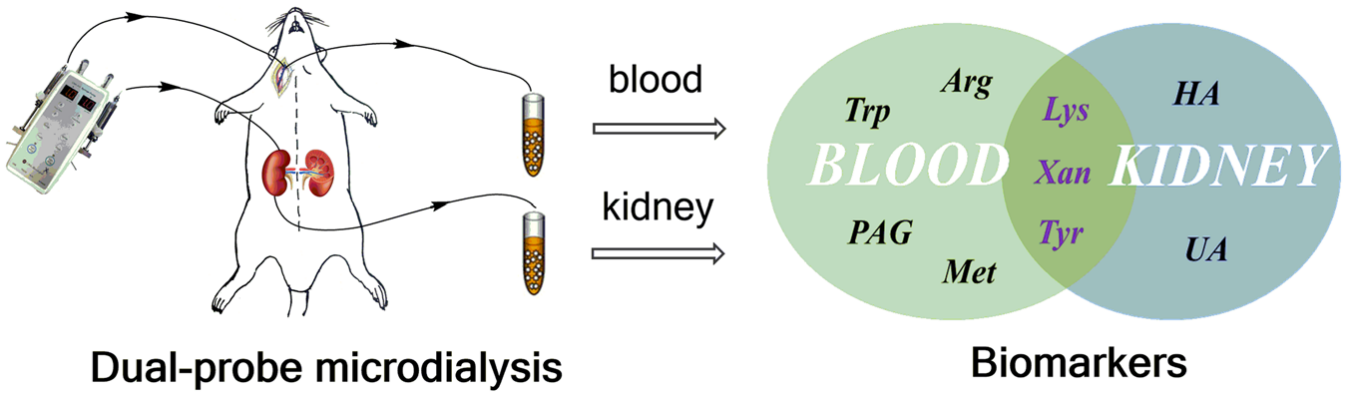
Potential therapeutic biomarkers in blood and kidney by dual-probe microdialysis sampling.

To monitor the treatment effects of *G. jasminoides* and discover potential therapeutic biomarkers, a partial least-square-discrimination analysis (PLS-DA) model was constructed using the data from blood and kidney dialysates. The score scatter plots for PLS-DA models from both kidney and blood dialysates showed clear differentiation of G-DM and DM groups (Fig. 5A and C). *R* ^2^ for the model and *Q* ^2^ for the prediction were 0.859 and 0.576 in blood and 0.997 and 0.948 in kidney tissues, respectively. The *R* ^2^ and *Q* ^2^ illustrated that the PLS-DA models were reliable. The variable importance in the projection (VIP) values were also applied to screen potential biomarkers. Using the VIP cut-off value (>1), the numbers of metabolites discriminating against the G-DM from the DM group were determined (Fig. 5B and D). In total, seven biomarkers in the kidney and five biomarkers in the blood were identified (Fig. 6).

To verify these biomarkers, PLS-DA model was performed using the data of E-DM and DM groups from blood and kidney dialysates, respectively. The score scatter plots for PLS-DA models from both kidney and blood dialysates showed clear differentiation of G-DM and DM groups (Supplementary Fig. S2A and C). *R* ^2^ for the model and *Q* ^2^ for the prediction were 0.954 and 0.835 in the blood and 0.996 and 0.974 in the kidney tissues, respectively. Similarly, six biomarkers in the kidney and four biomarkers in the blood were identified (Supplementary Fig. S2B and D). Compared with G-DM vs. DM model, E-DM vs. DM model has less biomarkers, and PAG is the unique biomarker for epalrestat treatment in the kidney. Other biomarkers are shared with G-DM vs. DM model, which probably imply similar therapeutic effects between *G. jasminoides* and epalrestat.

**Figure 7:**
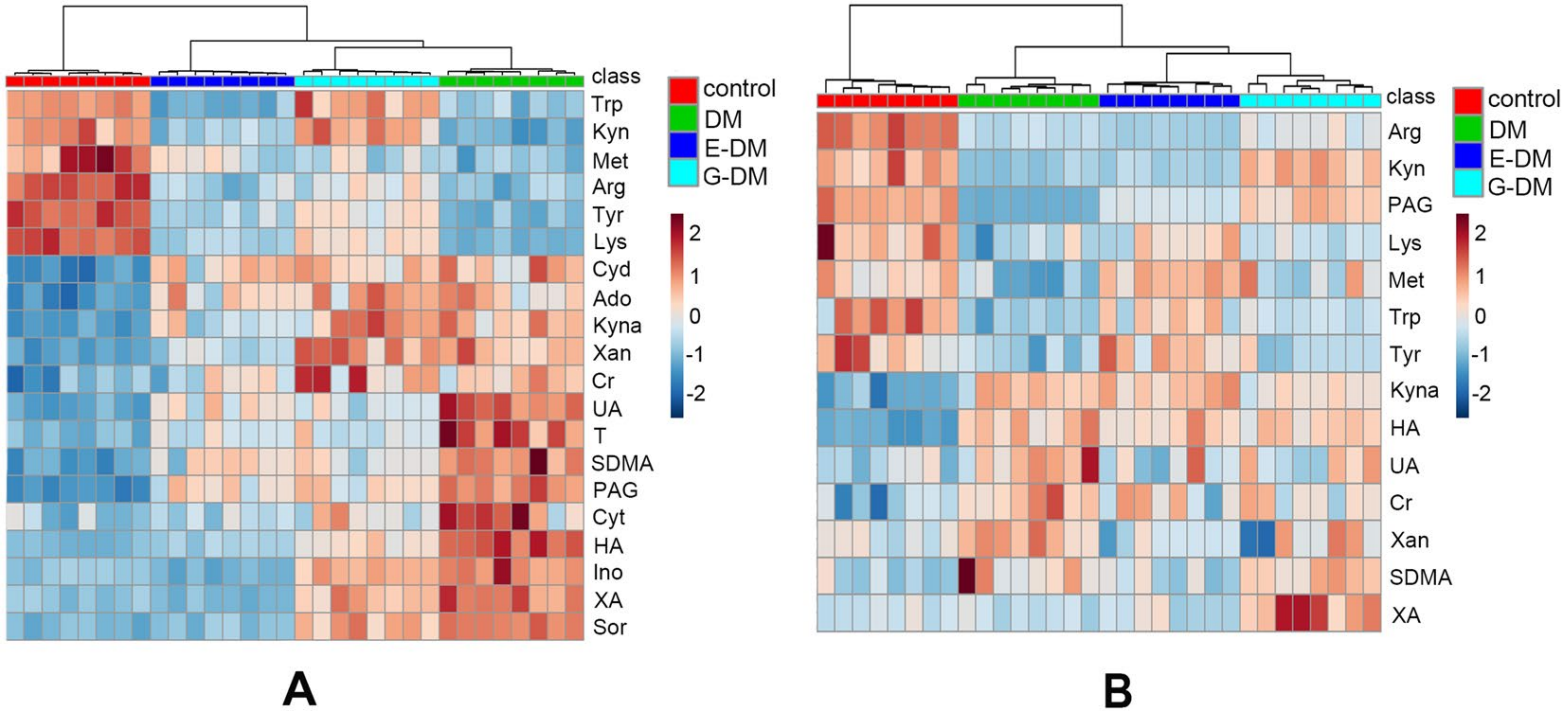
Heat maps of normalized metabolite concentrations in (**A**) kidney and (**B**) blood dialysates. Columns represent the samples (control, DM, E-DM, and G-DM groups), and rows represent the metabolites. The data were already normalized by using mean-centered.

Hierarchical clustering is technique in pattern recognition. Agglomerative hierarchical clustering begins with each sample as separate cluster and then proceeds to combine them until all the samples belong to one cluster. Results are presented as heat maps (Fig. 7). Overall, the model types do group together based upon the metabolite expression patterns of the blood and kidney dialysate samples, which can be seen in the clustering on the top of Fig. 7. Furthermore, metabolite patterns separated the four model types (control, DM, E-DM, and G-DM groups, marked with different colors) in blood dialysates as well as in kidney dialysates.

### Metabolic pathway analysis

Metabolic pathway analysis (MetPA) (http://metpa.metabolomics.ca) is a web-based metabolomics tool used to identify relevant pathways that are most likely to be associated with the condition under study. The impact factor of pathways analysis with MetPA was applied to evaluate the importance of the pathways on the development of diabetes in this study. Supplementary Table S4 shows eight disturbed metabolic pathways as the most relevant pathways involved in T2D. Obviously, five disturbed metabolic pathways among them were regulated by treatment with *G. jasminoides*. Results suggested that the therapeutic mechanism of *G. jasminoides* involved amino acid and purine metabolism.

**Figure 8:**
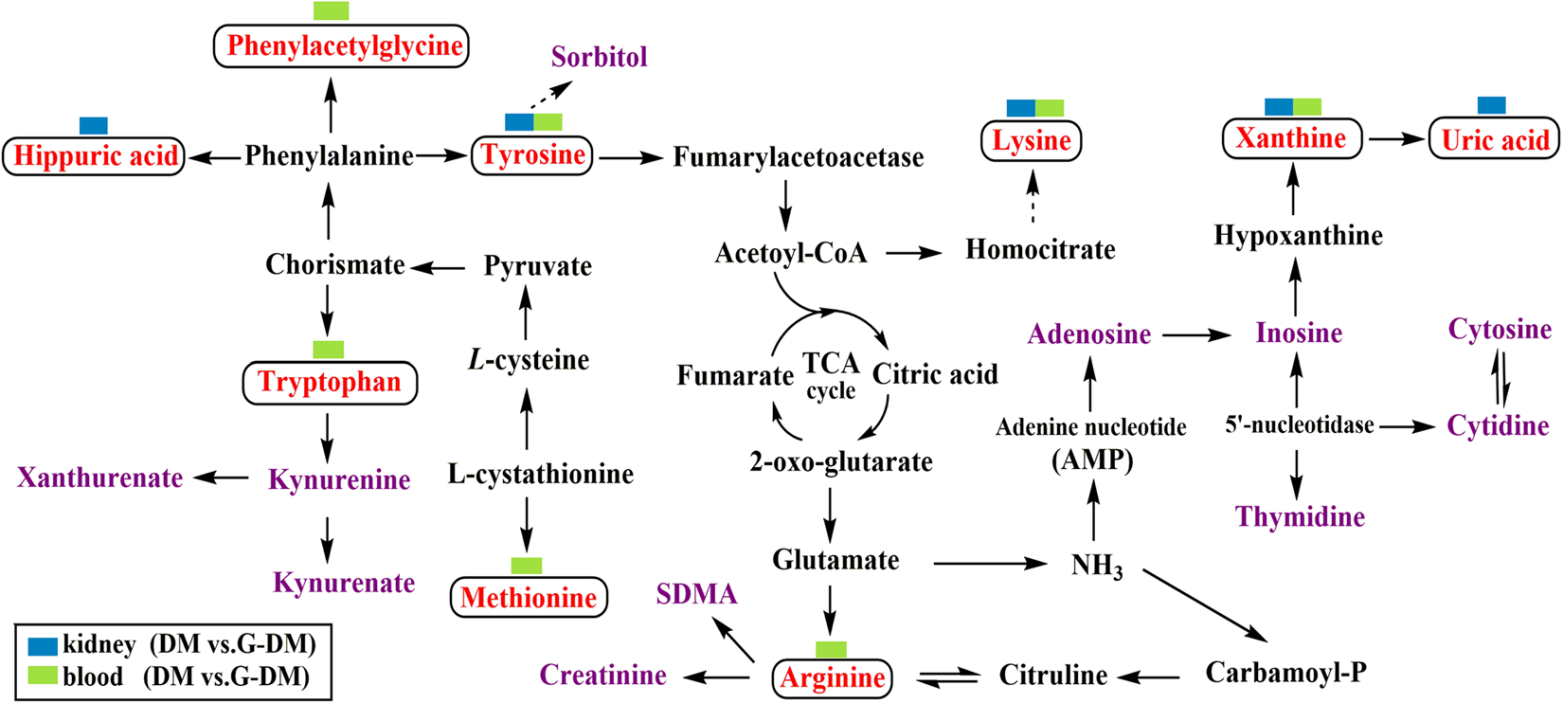
Correlation networks of main potential biomarkers in response to the therapeutic effects of *G. jasminoides* on diabetic rats. The potential biomarkers (VIP > 1) are marked as red; one rectangular strip above the potential biomarker contains two colors corresponding to blood and kidney.

In addition, the correlation networks of the main potential biomarkers of diabetes and the therapeutic effects of *G. jasminoides* on diabetic rats are intuitively described in Fig. 8. On top of the potential biomarkers, one rectangular strip is composed of different colors to represent the different groups, as illustrated in the legend.

## Discussion

### Tryptophan metabolism

In our study, a significant lower level of tryptophan (*p* < 0.01) was found in diabetic rats. For xanthurenate and kynurenate, concentrations were shown to be significantly higher (*p* < 0.01) in the blood and kidney tissues of diabetic rats than the normal rats. Tryptophan is an essential amino acid that can be metabolized through methoxyindole and kynurenine pathways. The majority (about 95%) of tryptophan is metabolized by the kynurenine pathway (KP)^24^, leading to the production of various metabolites, including kynurenate and xanthurenate. The key enzymes of tryptophan to kynurenine conversion are tryptophan 2,3-dioxygenase (TDO) and indoleamine 2,3-dioxygenase (IDO), which are activated by stress hormones or inflammatory factors. Previous study suggested the diabetogenic effects of KP metabolites, such as xanthurenate-induced hyperglycemia^25^; the increased urine excretion of xanthurenate in rat model of diabetes^26^; and the biological activity of insulin by kynurenine, xanthurenate, kynurenate, and their derivatives^27^. Thus, overproduction of diabetogenic kynurenine, xanthurenate, and kynurenate was one of the mechanisms of chronic stress- or chronic low-grade inflammation-induced development of T2D^28^. The increased urine excretion of xanthurenate was reported in 3 pre-diabetic^29^ and 20 T2D patients^26^ which were consistent with our findings. Kynurenine/tryptophan ratio is frequently used as an indicator of IDO activity^30^. In the blood, kynurenine/tryptophan ratio increased in DM models compared with normal rats. The results show that TDO is probably activated by stress hormones or inflammatory factors in diabetic rats. However, no increase in kynurenine/tryptophan ratio in DM models was observed in kidney tissues, suggesting that other mechanisms might be involved.

### Arginine metabolism

Increased SDMA and creatinine along with decreased levels of arginine were observed in diabetic rats in our study. Nitric oxide (NO) is generated from arginine by the enzyme synthase (NOS). Previous study showed that the physiological levels of NO played an important role in regulating the oxidation of metabolic intermediates, insulin sensitivity, and hemodynamics in animals and humans^31^. During arginine depletion, NOS generated reactive oxygen species (ROS) in lieu of NO and can thus increase oxidative stress^32^. Oxidative stress is widely accepted as being associated with T2D. SDMA and its stereoisomer asymmetric dimethylarginine (ADMA) are produced during the post-translational methylation of arginine residues by protein arginine methyl transferases (PRMTs) in proteins^33^. SDMA was fully eliminated through the kidney and strongly related to renal function^34^, including creatinine clearance^35^. Further study indicated that the plasma levels of SDMA were significantly higher (*p* < 0.01) in patients with type 1 diabetes (T1D) based on the controls^35^. The results are in agreement with our findings.

### Lysine metabolism

The observed reduction of lysine in diabetic rats was related to the mechanisms below. Research in humans and rodents has indicated that lysine potentiate glucose stimulated insulin secretion. Lysine ingestion resulted in a small decrease in serum glucose and an increase in glucagon and insulin concentrations^36, 37^. In addition, the reversible acetylation of lysine in an organ led to the long-term complications of diabetes, including DN^38^.

### Phenylalanine metabolism

In our study, the levels of tyrosine are lower and the levels of hippuric acid are higher in diabetic rats. Phenylalanine and tyrosine are aromatic amino acids (AAAs). Studies corroborated results that linked increases in the AAAs with obesity and T2D^39, 40^. Tyrosine is a semi-essential amino acid that is only synthesized by the hydroxylation of phenylalanine by the enzyme phenylalanine hydroxylase. Chronic renal impairment was associated with decreased plasma concentrations of tyrosine^41^. Similarly, in our study, tyrosine depletion in diabetic rats was associated with an increased risk of having renal impairment. Hippuric acid is produced in the gut by microorganisms using glycine and benzoic acid as building blocks^42^. Phenylacetate was transformed from phenylalanine through the action of gut microbiota; phenylacetate was combined with glycine to form phenylacetylglycine^43^. The notion that intestinal microbiota as a key player in the onset and development of diabetes is becoming more widely accepted. Higher levels of some bacterial metabolites have been detected in urine samples of diabetic rats^44^. Furthermore, an important role of the gut in the pathogenesis of T1D has been suggested, due not only to the intestinal microbiota but also to altered intestinal permeability and immunity^44, 45^.

### Purines and pyrimidines metabolism

The significant higher levels of uric acid and xanthine (*p* < 0.01) were observed in diabetic rats. Purines and pyrimidines are the basic constituents of DNA and RNA. Purine and pyrimidine metabolism is associated with the development of diabetes and DN^19^. During purine metabolism, hypoxanthine is accumulated and is further metabolized to xanthine and uric acid by xanthine dehydrogenase or xanthine oxidase, along with the generation of ROS. High serum uric acid (SUA) is associated with the increased risk of the development of chronic kidney disease in T2D^46^. In addition to chronic kidney disease, some studies reported that the elevated levels of SUA were associated with hypertension, coronary heart disease, and stroke^47, 48^.

### Polyol pathway

The levels were found to be significantly higher (*p* < 0.01) in diabetic rats in our study. Sorbitol occurs naturally and is also produced synthetically from glucose. Excessive sorbitol seriously blocks the cell membrane pervasion and ultimately leads to chronic complications of diabetes.

In summary, we applied dual-probe microdialysis combined with liquid chromatography coupled to a triple quadrupole tandem mass spectrometer, which enables the qualitative and quantitative analysis of endogenous metabolites, simultaneously to measure their levels in the kidney tissues and blood obtained from T2D rats. Results indicated that the change trends of metabolites in the blood and kidney tissues were highly synchronized. As we known, blood passes through the glomeruli and forms the original urine and renal tubule reabsorbs and continues to separate substances from the original urine in kidney, thus making the constituents in the kidney tissues parallel to the blood. Amino acid levels were lower, but the nucleotide metabolite levels were higher in diabetic rats compared with those in the normal rats. Targeted metabolomics analysis showed the global metabolic alterations of diabetic rats before and after *G. jasminoides* treatment. Furthermore, potential therapeutic biomarkers were discovered, which were related to multiple biochemical processes, such as phenylalanine, tyrosine, and tryptophan biosynthesis, tryptophan metabolism, tyrosine metabolism, arginine and proline metabolism, and purine metabolism. Among them, the metabolic perturbations of phenylalanine, tyrosine, and tryptophan biosynthesis; tryptophan metabolism; and purine metabolism were regulated after *G. jasminoides* treatment in kidney tissues. Results provided a unique perspective on localized metabolic information of T2D and its complications, which gave us new insights into the pathogenesis of diabetes as well as the discovery of targets for clinical diagnosis and treatment.

## Methods

### Reagents and chemicals

Sorbitol (Sor); xanthine (Xan); inosine (Ino); adenosine (Ado); uric acid (UA); thymidine (T); cytidine (Cyd); cytosine (Cyt); arginine (Arg); symmetric dimethylarginine (SDMA); creatinine (Cr); tryptophan (Try); kynurenine (Kyn); kynurenate (Kyna); xanthurenate (XA); phenylacetylglycine (PAG); hippuric acid (HA); tyrosine (Tyr); methionine (Met); lysine(Lys); N, N-dimethylphenylalanine (N, N-Phe); and streptozotocin (STZ) were purchased from Sigma-Aldrich (USA). HPLC-grade methanol, formic acid, and acetic acid were purchased from Fisher Scientific (USA). Water was obtained from Millipore Milli-Q Ultra-pure water purification system (Belgium). Ringer’s solution (145 mM Na^+^, 1.2 mM Ca^2+^, 4 mM K^+^, and 0.1 mM ascorbic acid pH 7.0) and anticoagulant citrate dextrose (ACD) solution (3.5 mM citric acid, 7.5 mM sodium citrate, and 13.6 mM dextrose) were prepared as perfusate for microdialysis. Epalrestat was obtained from the Yangtze River Pharmaceutical Group (Taizhou, China). The extract of *G. jasminoides* had been prepared and identified in our previous study^49^.

### Animal experiment

Male Sprague–Dawley rats (180–200 g) were obtained from the Experimental Animal Center of Jilin University (Jilin, China). The rats were bred in a 12 h light and 12 h dark cycle with regulated temperature (23 ° ± 2 °C) and humidity (40%–80%). Animal welfare and experimental procedures were approved by the Animal Research Ethics Committee of Jilin University and all methods were performed in accordance with the relevant guidelines and regulations. After one-week adaptation, the rats were randomly divided into four groups: control group, diabetes model (DM) group, E-DM group, and G-DM group. The control group received a standard diet. The other groups were fed with high-fat and high-sucrose chow (18% lard, 3% cholesterol, 20% sucrose, and 59% standard rat chow) as DM group. After 10 weeks of feeding, diabetic rats were given STZ (35 mg/kg BW in citrate buffer)^50^. Rats presenting blood glucose levels lower than 16.7 mmol·L^−1^ were excluded from the diabetic rat group. After the confirmation of diabetes, the E-DM group (positive control) and G-DM group received an orally administered epalrestat (0.35 mg/kg BW) and *G. jasminoides* (4.4 g/kg BW) via a gastric tube once a day for another 12 weeks. The control group and DM group were administered with distilled water 2 mL for 12 weeks.

### Dual-probe MD of blood and kidney tissues

Blood and kidney microdialysis systems consisted of a microdialysis syringe pump (CMA/402, CMA, Stockholm, Sweden), microdialysis probes, and a stereotaxic frame. The dialysis probes (Microbiotech AB, Stockholm, Sweden) for the blood (molecular weight cut-off of 20 kDa) and kidney (molecular weight cut-off of 6 kDa) were 10 mm in length.

On the day of microdialysis, the probes were perfused with Ringer’s solution at a constant flow rate (1 μL·min^−1^) by using a micro-infusion pump. The animals were initially anesthetized with sodium pentobarbital (40 mg/kg) and immobilized in a supine position. The blood microdialysis probe was inserted into the jugular vein to the right atrium. The animals were placed in the lateral decubitus position prior to the surgical removal of the skin and muscle. The linear microdialysis probe was inserted through the kidney medulla with the aid of a needle, which was removed after insertion. Then, two probes were perfused with ACD solution and Ringer’s solution at a flow rate of 1 μL·min^−1^, respectively. After the stabilization period, the dialysates from the blood and kidney tissues were collected at 30 min intervals for 3 h into the fraction collector (MAB 85). The dialysates were preserved at −80 °C before analysis.

### LC-MS/MS conditions

LC–MS/MS analysis was performed with a Waters ACQUITY UPLC system (Waters Corp., Milford, MA, USA) connected to a Xevo TQ MS spectrometer with an electrospray ion source (ESI) (Waters, Manchester, UK). Chromatographic separation was carried out on a Waters UPLC BEH Shield RP18 column (100 mm × 2.1 mm, 1.7 μm) using gradient elution. The mobile phase was composed of acetonitrile (A) and water containing 0.1% (v/v) formic acid (B). The injected volume was 5 μL, and a flow rate of 200 μL·min^−1^ was applied during the gradient elution. The elution program was as follows: 0–3 min, 1% A (isocratic); 3–9.5 min, 1%–40% A (linear increase); 9.5–12.5 min, 40%–80% A (linear increase); 12.5–13 min, 80%–100% A (linear increase); 13–16 min, 100% A (isocratic); 16–18 min, 100%–10% B (linear decrease); and 18–19 min, 10%–1% A (linear decrease). Data were acquired in positive ion mode. The source parameters for the MS were set as follows: a capillary voltage of 3.0 kV; source temperature, 150 °C; desolvation temperature, 350 °C; desolvation gas flow, 800 L·h^−1^; and cone gas flow, 50 L·h^−1^. Nitrogen was used as the cone and desolvation gas, and argon was used as the collision gas. Multiple reaction monitoring (MRM) transitions for 21 analytes, as well as their respective cone voltages and collision energies, are listed in Supplementary Table S5.

### *In vitro* relative recovery

To measure *in vitro* recovery, blood and kidney probes were perfused at a flow rate of 1 μL·min^−1^ and placed in a stirred vial containing all standards in Ringer’s solution. The relative recovery (RR) was calculated as follows:

1 $${\rm{RR}}( \% )=({{\rm{C}}}_{out}/{{\rm{C}}}_{in})\times 100$$

where *C*_*out*_ is the ratio between the concentrations in the dialysate; *C*_*in*_ is the concentration in the medium surrounding the probe^51^.

The average values of *in vitro* relative recovery of blood and kidney probes for 20 analytes are listed in Supplementary Table S1. The *in vitro* RR values could be affected by the temperature and perfusion rate. In addition, *in vitro* RR values are dependent on the nature of analytes and the tissue investigated, and these values were used to correct analyte concentrations determined in kidney and blood of rats.

### Method validation and endogenous metabolite quantitation

Method validation has been shown in Supplementary Method validation.

MD technique enables the direct detection of endogenous compounds such as metabolites without sample preparation, particularly for tissue sampling. More importantly, MD is a well-established *in vivo* sampling method that may cause minimal perturbation to biological systems. *In vivo* dual-probe MD combined with LC-MS/MS, which enables the quantitative analysis of endogenous metabolites, was applied to simultaneously measure metabolite levels in the blood and kidney tissues of rats. Under optimized conditions, the MRM transitions in positive mode are shown in Supplementary Fig. S1. The above developed method was applied to analyze the blood and kidney tissue dialysates obtained from rats. The extracted ion chromatograms of dialysates in kidney tissues indicated that all analytes were identified and quantified in a single LC-MRM-MS run. The levels of endogenous metabolites were calculated based on their respective calibration curves (Supplementary Table S2) and relative recovery values (Supplementary Table S1). Then, their corresponding concentration levels in the blood and kidney tissues are shown in Fig. 1 and Fig. 2.

### Biochemical analyses (shown in Supplementary Biochemical analyses online)

#### Data processing and statistical analysis

LC-MS/MS data were acquired using the MassLynx V4.1 software and processed for the calibration and quantification of the analytes using the Targetlynx V4.1 software. Statistical analysis was managed with SPSS 18.0 version software, and *p* < 0.05 was considered to be a significant difference for Student’s t test.

PLS-DA, PCA, and heat maps were generated by using MetaboAnalyst 3.0 (http://www.metaboanalyst.ca.)^52^. Data were mean-centered and divided by the square root of standard deviation of each variable.

## Electronic supplementary material

Supplementary information
