# Supplementary material for: Targeted metabolome profiling by dual-probe microdialysis sampling and treatment using Gardenia jasminoides for rats with type 2 diabetes
Source: Sci Rep. 2017 Aug 31;7:10105. doi: 10.1038/s41598-017-10172-w (PMC5579158; doi:10.1038/s41598-017-10172-w)
Supplement: Supplementary file 1 — Supplementary information [file 41598_2017_10172_MOESM1_ESM.pdf]

Supplementary Information

Targeted metabolome profiling by dual-probe microdialysis sampling and treatment using *Gardenia jasminoides* for rats with type 2 diabetes

Lu Wang[1,2†], Zifeng Pi[1], Shu Liu[1], Zhiqiang Liu[1], Fengrui Song[1*]

[1] *National Center of Mass Spectrometry in Changchun, Jilin Province Key Laboratory of Chinese Medicine Chemistry and Mass Spectrometry, Changchun Institute of Applied Chemistry, Chinese Academy of Sciences, Changchun 130022, China*

[2]*University of Chinese Academy of Sciences, Beijing 100039, China*

* Corresponding authors:

Tel: +86-431-85262044; Fax: +86-431-85262044; E-mail:songfr@ciac.ac.cn (F.Song)

CONTENTS

Supplementary Method validation

Supplementary Figure S1-S2

Supplementary Table S1-S6

Method validation

The analytical performance parameters were evaluated including linearity, limit of detection (LOD) and the limit of quantification (LOQ), precision (intra and inter-assay), accuracy and matrix effect. A stock solution containing the analytes was diluted with a solution of Ringer's to prepare a series of working standard solutions for calibration and quality control (QC) samples. Calibration curves for analytes were constructed using the peak areas of the quantification MRM transitions against the theoretical concentrations. The LOD and LOQ were measured based on the signal-to-noise ratios of 3:1 and 10:1, respectively. The intra- and inter-day precisions were evaluated by assessing six replicate QC samples in Ringer's solution at three different concentrations on two consecutive days. The relative error (RE) of each QC point served as the measure of accuracy. The post-extraction spike method[18] was adopted in our study to evaluate the matrix effect (ME) that was calculated as follows:

$$\mathrm{ME}(\%) = \frac{B-C}{A} \times 100 \qquad \text{Equation (2)}$$

The peak areas obtained in neat solution standards were depicted as A, the corresponding peak areas for standards spiked into dialysate as B, and peak areas for analytes in non-spiked dialysate as C.

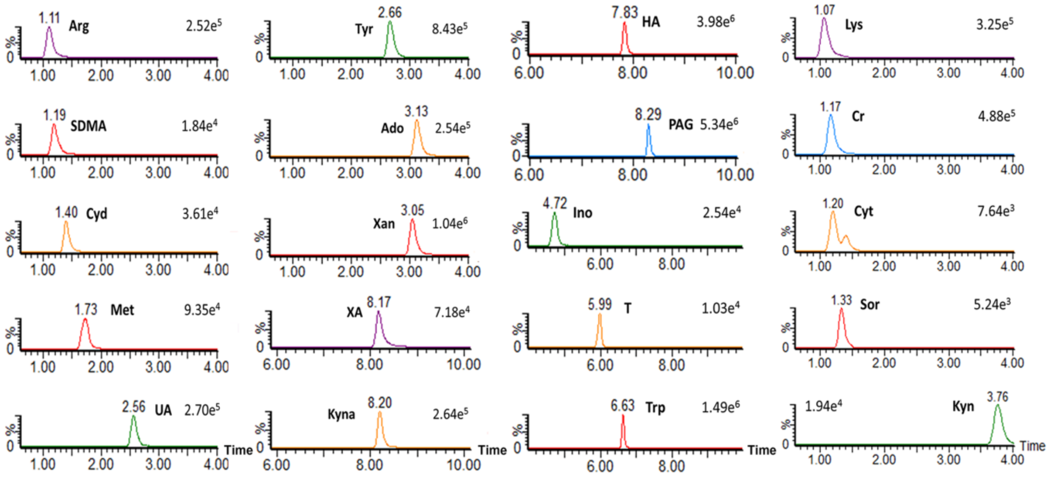

Supplementary Figure S1 Extracted ion chromatograms of 20 analytes in kidney dialysates of diabetes rats

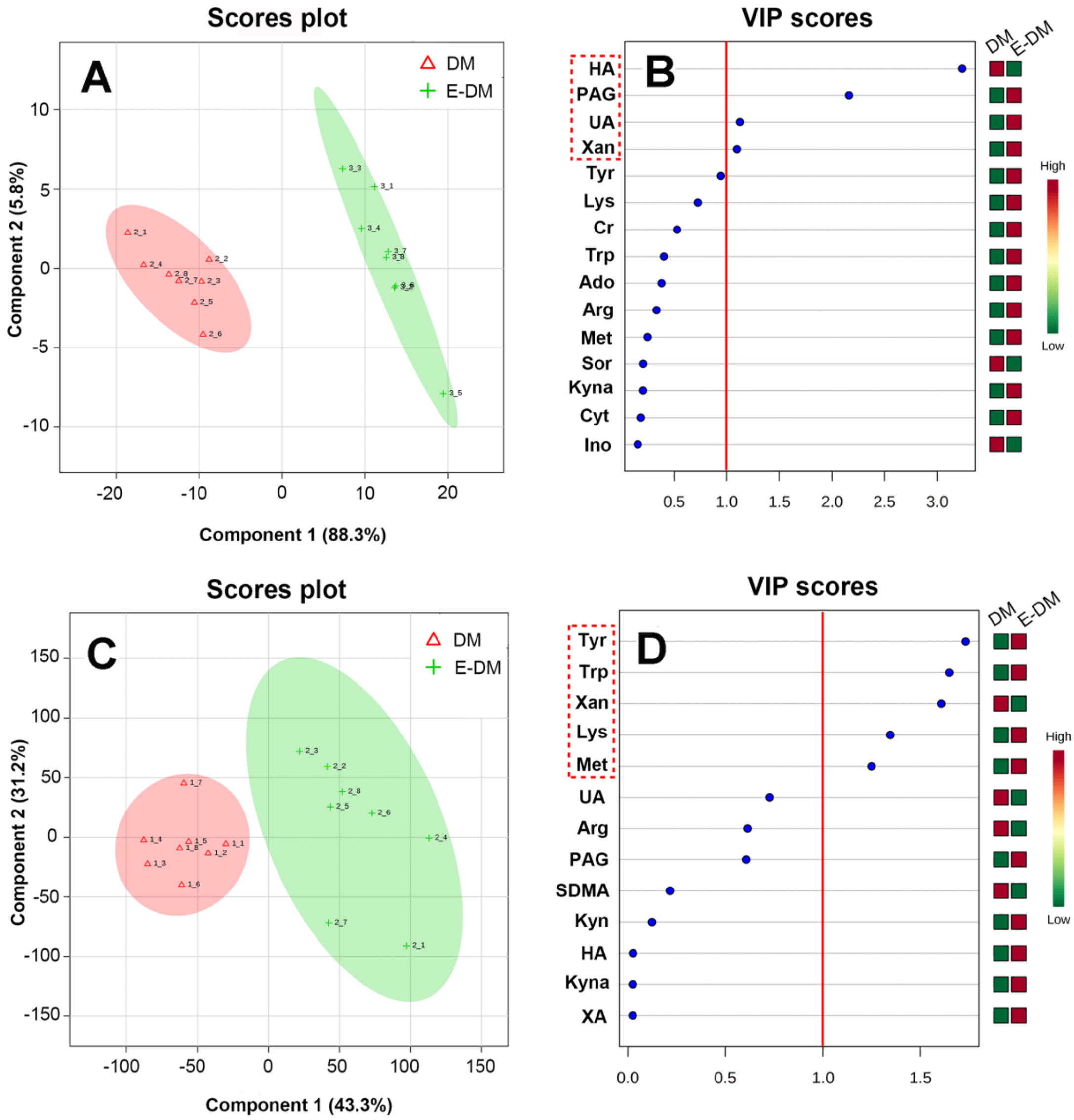

Supplementary Figure S2 PLS-DA score plots showing a significant separation between E-DM and DM groups in the kidney (A) and blood (C); VIP scores representing the most contributing metabolites involved in the separation between E-DM and DM groups in the kidney (B) and blood (D).

Supplementary Table S1 Relative recoveries of blood and kidney probes

**Analytes**	**RR (%)**	
	**Probe 1(blood)**	**Probe 2(kidney)**
Sorbital	27.96±0.66	27.23±0.78
Creatinine	30.93±4.99	28.21±1.06
Lysine	28.14±1.25	22.56±1.02
Methionine	24.24±3.80	25.99±1.60
Xanthine	34.91±1.17	23.25±2.37
Uric acid	29.55±1.29	27.64±0.82
Arginine	20.41±0.83	23.40±1.00
Tyrosine	35.67±1.61	21.15±1.99
Cytosine	25.12±3.78	23.35±4.88
SDMA	31.32±1.07	19.80±4.79
Kynurenine	28.80±4.44	17.57±0.24
Cytidine	20.39±3.94	18.53±3.88
adenosine	13.89±0.72	20.67±1.96
Hippuric acid	22.99±3.66	15.34±5.19
Kynurenate	27.70±4.60	16.24±0.43
Phenylacetylglycine	20.91±2.07	17.41±4.99
Xanthurenate	29.99±4.34	24.41±0.10
Tryptophane	29.83±3.17	26.54±0.94
Thymidine	26.98±4.02	20.42±0.90
Inosine	39.02±2.55	28.51±4.04

Supplementary Table S2 Linearity, LODs and LOQs of 20 analytes

**Analytes**	**Dynamic range** (ng/mL)	**Slope** mean ±SD	***Y*-intercept** mean ±SD	**R^2**	**LODs** (ng/mL)	**LOQs** (ng/mL)
Creatinine	20-1000	0.4007 ±0.0007	2.118 ±0.205	0.9993	0.25	0.5
Methionine	10-500	0.5438 ±0.0016	-2.455 ±0.297	0.9987	0.5	2
Xanthine	200-10000	0.1977 ±0.0021	41.79 ±2.66	0.9985	1	5
Uric acid	100-5000	0.0927 ±0.0022	9.729 ±1.461	0.9971	0.5	2
Hippuric acid	10-500 [a]	0.9976 ±0.0014	-8.241 ±0.651	0.9973	0.25	0.5
	500-20000 [b]	0.5585 ±0.0071	511.9 ±15.6	0.9958	0.25	0.5
Phenylacetylglycine	1.0-10000 [a]	1.1192 ±0.0031	-1.306 ±0.101	0.9995	0.075	0.25
	500-10000 [b]	0.8022 ±0.0023	349.2 ±9.1	0.9982	0.075	0.25
Arginine	10-500	1.0020 ±0.0005	-0.7046 ±0.6180	0.9975	0.1	0.5
Tyrosine	100-5000	0.3465 ±0.0087	10.38 ±4.33	0.9993	0.1	0.25
Sorbitol	10-500	0.0425 ±0.0000	-0.286 ±0.150	0.9973	0.075	2
SDMA	2.0-100	0.2503 ±0.0072	-0.5304 ±0.1095	0.9996	0.25	0.5
Lysine	100-5000	0.1764 ±0.0025	43.13 ±1.63	0.9920	0.1	1
Kynurenine	2.0-100	0.50130.0084	-1.046 ±0.105	0.9986	0.1	0.5
Cytidine	1.0-50	0.4545 ±0.0002	-0.2754 ±0.0099	0.9969	0.1	0.5
Adenosine	20.0-500	1.2734 ±0.005	-7.247 ±0.243	0.9996	0.25	0.5
Kynurenate	2.0-100	2.3467 ±0.0016	0.2536 ±0.1320	0.9969	0.1	0.5
Xanthurenate	5.0-200	1.120.0371	-1.646 ±1.233	0.9996	0.05	0.1
Tryptophane	100-2000	0.9003 ±0.0264	19.55 ±12.59	0.9966	0.5	5
Thymidine	5.0-200	0.0727 ±0.0013	-0.235 ±0.076	0.9987	1	5
Inosine	5-100	0.3672 ±0.0005	-0.0993 ±0.0503	0.9956	0.25	2
Cytosine	20-500	0.0741 ±0.0002	-1.444 ±0.076	0.9970	0.25	10

[a]dynamic range of blood dialysates ; [b]dynamic range of kidney tissue dialysates

Supplementary Table S3 Summary of precision, accuracy and matrix effect

Analytes	Added ng/mL	Intra-day			Inter-day			Matrix effect% ME%
		Observed	Precision RSD%	Accuracy RE%	Observed	Precision RSD%	Accuracy RE%	
Creatinine	20	20.68±1.94	9.38	3.38	21.39±2.58	10.79	1.13	93.32±5.90
	100	97.10±4.42	4.56	-2.90	93.59±4.52	4.84	-6.41	96.24±9.05
	500	486.07±4.45	0.92	-2.79	482.21±27.09	5.62	-3.56	89.20±2.18
Methionine	20	21.09 ±0.70	3.33	5.43	20.89 ±0.83	3.97	4.43	88.16±4.74
	100	93.86 ±5.79	6.17	-6.14	94.20 ±6.27	6.65	-5.80	88.85±0.75
	500	487.30 ±34.29	7.04	-2.54	490.64 ±36.29	7.40	-1.87	96.72±1.78
Xanthine	500	448.44±18.97	4.23	-10.31	445.08±41.00	9.21	-10.98	88.17±2.15
	2000	2145.10±51.76	2.41	7.26	2145.56±72.89	3.40	7.28	92.15±1.34
	10000	9791.74±214.88	2.19	-0.78	9980.70±327.66	3.28	-0.91	103.00±0.92
Uric acid	500	500.25±5.37	1.07	0.05	504.01±29.34	5.82	0.80	103.54±2.09
	1000	1078.61±63.04	5.84	7.86	1100.20±47.93	4.32	10.02	104.13±2.55
	5000	4982.60±202.94	4.07	-0.35	4945.23±203.30	4.11	-1.10	102.85±2.68
Hippuric acid	100	95.96±3.91	4.07	-4.04	92.97±4.65	5.00	-7.03	108.97±3.72
	500	470.54±13.54	2.88	-5.89	493.49±25.25	5.12	-1.30	105.86±1.17
	10000	11266.95±502.52	4.46	12.67	10997.74±498.72	4.53	9.98	97.95±1.04
Phenylacetylglycine	20	21.08±0.95	4.50	5.39	19.75±1.94	9.84	-1.23	99.20±5.28
	1000	1066.00±34.31	3.22	6.60	984.93±115.15	11.69	-1.51	96.97±0.78
	5000	5358.83 ±143.97	2.69	7.18	5323.00±154.37	2.90	6.46	100.12±1.74
	50	52.52 ±4.91	9.35	5.03	52.02 ±5.46	10.49	4.03	88.12±1.98

Arginine	200	191.48 ±11.88	6.20	-4.26	186.99 ±11.88	6.35	-6.50	91.87 ±1.03
	500	473.97 ±31.00	6.54	-5.21	481.61 ±33.45	6.95	-3.68	88.88 ±1.72
	200	180.28 ±2.61	1.45	-9.86	202.56 ±8.58	4.24	1.28	102.83 ±4.36
Tyrosine	1000	977.82 ±22.01	2.25	-2.22	1065.33 ±84.83	7.96	6.53	94.67 ±0.63
	5000	5063.54 ±70.05	1.38	1.27	4680.39 ±155.62	3.32	-6.39	98.51 ±0.83
	50	53.60 ±4.47	8.33	7.21	50.11 ±5.89	11.76	0.22	92.34 ±8.54
Sorbital	100	94.13 ±1.12	1.19	-5.87	94.83 ±4.67	4.93	-5.17	88.76 ±1.98
	200	184.61 ±6.49	3.51	-7.69	193.44 ±11.54	5.96	-3.28	93.25 ±4.80
	10	10.91 ±0.69	6.33	9.09	11.15 ±0.96	8.63	11.47	90.20 ±8.69
SDMA	50	54.82 ±4.30	7.84	9.63	53.72 ±5.74	10.68	7.63	96.93 ±4.04
	100	108.89 ±1.29	1.18	8.89	105.68 ±6.17	5.84	5.68	95.88 ±4.08
	200	200.38 ±17.85	8.91	0.19	192.60 ±21.31	11.07	-3.70	91.77 ±3.71
Lysine	500	462.45 ±32.09	6.94	-7.51	480.19 ±34.35	7.15	-3.96	96.39 ±2.75
	2000	2184.38 ±132.64	6.07	9.22	2197.61 ±143.80	6.54	9.88	92.74 ±1.03
	10	8.88 ±0.44	9.42	-11.18	8.94 ±0.52	5.87	-10.57	103.55 ±11.02
Kynurenine	20	19.82 ±0.09	0.43	-0.89	18.93 ±1.64	8.56	-5.36	96.94 ±9.35
	50	54.43 ±1.45	2.66	8.86	50.73 ±3.06	6.04	1.05	95.69 ±2.93
	5	4.49 ±0.04	0.90	-10.15	4.68 ±0.07	1.52	-6.34	91.94 ±1.83
Cytidine	10	9.64 ±0.49	5.13	-3.57	9.15 ±0.53	5.77	-8.05	99.42 ±6.89
	20	20.65 ±1.57	7.60	3.24	19.69 ±2.45	12.46	-1.55	89.35 ±2.00
	50	52.91 ±1.98	3.74	5.81	53.51 ±2.10	3.93	7.02	105.44 ±4.50
Adenosine	100	99.69 ±3.74	3.75	-0.31	98.42 ±4.18	4.25	-1.58	99.45 ±2.17
	200	201.13 ±8.17	4.06	0.56	199.58 ±9.56	4.79	-0.21	104.31 ±2.84
	5	4.88 ±0.46	9.49	-2.37	4.77 ±0.52	10.80	-4.55	93.99 ±4.08
Kynurenate	20	20.07 ±1.24	6.18	0.34	19.43 ±1.86	9.59	-2.85	99.72 ±4.69

	100	104.42±5.95	5.70	4.42	106.01±7.12	6.71	6.01	99.30±0.98
	5	5.18±0.24	4.58	3.60	5.54 ±0.70	12.61	10.82	99.66±9.28
Xanthurenate	100	111.96±3.78	3.38	11.96	110.09±5.95	5.41	10.09	97.53±2.36
	200	216.41±9.11	4.21	8.21	213.17 ±9.02	4.23	6.59	100.31±2.36
	200	192.77 ±11.66	6.05	-3.62	189.48 ±12.49	6.59	-5.26	98.89±1.15
Tryptophane	500	460.19 ±8.52	1.85	-7.96	487.25 ±39.40	8.09	-2.55	96.79±0.56
	1000	976.94 ±45.89	4.70	-2.31	1047.37 ±101.78	9.72	4.74	95.24±0.96
	20	20.96±0.11	0.52	4.79	20.46±1.15	5.62	2.30	88.28±9.66
Thymidine	100	94.92±8.09	8.53	-5.08	94.28±10.84	11.50	-5.72	94.87±6.13
	200	186.29±3.28	1.76	-6.85	186.31±7.15	3.84	-6.84	93.31±4.53
	20	19.97±0.99	4.97	-0.13	19.47±1.49	7.56	-2.67	102.30±5.04
Inosine	50	53.43±0.55	1.02	6.87	53.31±4.00	7.51	6.61	105.49±4.13
	100	100.95±5.14	5.09	0.95	106.67±6.04	5.66	6.67	107.11±4.23
	50	50.62 ±3.32	6.57	1.23	50.57 ±4.07	8.05	1.14	93.89±9.07
Cytosine	100	99.10 ±6.43	6.49	-0.90	99.45 ±7.99	8.03	-0.55	89.98±4.74
	200	183.76±16.25	8.84	-8.12	194.45 ±17.62	9.06	-2.77	91.81±8.25

Supplementary Table S4 The main disturbed metabolic pathways

Pathways name	Impact			
	kidney		blood	
	Control vs. DM model	G-DM vs. DM model	Control vs. DM model	G-DM vs. DM model
Phenylalanine, tyrosine and tryptophan biosynthesis	0.5	0.5	0.5	0.5
Tryptophan metabolism	0.2870	-	0.2870	0.1772
Tyrosine metabolism	0.1405	0.1405	0.1405	0.1405
Cysteine and methionine metabolism	0.0869	-	0.0869	-
Arginine and proline metabolism	0.0823	-	0.0823	0.0823
Pyrimidine metabolism	0.0619	-	-	-
Purine metabolism	0.0573	0.0537	0.0537	0.0329
Fructose and mannose metabolism	0.0314	-	-	-

Supplementary Table S5 Multiple reaction monitoring (MRM) parameters for 20 analytes

Analytes	Cone voltage(V)	Quantitation transition (m/z)	Confirmation transition (m/z)	Function
		Collision energy[a]（eV）	Collision energy[a]（eV）	
Cytosine	18	112.09>95.01 (25)	112.09>52.05 (25)	1
Creatinine	20	114.03>86.09 (10)	114.03>44.14 (14)	1
Lysine	16	147.09>84.13(16)	147.09>56.1 (26)	1
Methionine	14	150.05>132.99 (10)	150.05>104.04 (14)	1
Xanthine	24	152.98>135.95 (21)	152.98>109.97 (18)	1
Uric acid	24	169.01>141.07 (16)	169.01>70.07 (22)	1
Arginine	20	175.03>70.13 (20)	175.03>60.11 (12)	1
SDMA	20	203.1>115.99 (18)	203.1>70.21 (26)	1
Tyrosine	18	182.02>165.02 (12)	182.02>136.01 (16)	1
Sorbital	10	183.01>129.04 (10)	183.01>69 (12)	1
Kynurenine	16	209.05>146.07 (20)	205.06>94.06 (16)	1
Cytidine	10	244.06>112.01 (8)	244.06>94.98 (40)	1
Adenosine	18	268.08>136.06 (18)	268.08>119.03 (42)	1
N,N-Phe（IS）	22	194.21>133.05 (26)	194.21>148.16 (16)	2
Tryptophane	14	205.01>146.04 (18)	205.01>118.01 (26)	2

Thymidine	10	243.01>126.96 (6)	243.01>109.98 (38)	2
Inosine	10	269.07>137.04 (16)	269.07>110.05 (40)	2
Hippuric acid	12	180.01>105.06 (18)	180.01>77.04 (24)	3
Kynurenate	24	190.01>144.06 (16)	190.01>116.11 (28)	3
Phenylacetylglycine	12	194.04>91.08 (20)	194.04>76.06 (10)	3
Xanthurenate	22	206.01>160.06 (20)	206.01>132.04 (28)	3

[a] The values of collision energy (eV) are given in the brackets.

Biochemical analyses

The renal function related parameters in control and DM groups were assayed. The concentrations of albumin in urine samples, serum creatinine (Scr) and urea nitrogen (BUN) were determined by the commercial assay kits (Nanjing Jiancheng, Institute of Biotechnology, Nanjing, China). And 24-h urinary albumin excretion rate (UAER) was calculated.

Supplementary Table S6 Renal functional parameters of rats in different groups

Group	UAER (mg/24h)	BUN (mmol/L)	Scr (μmol/L)
Control	4.32±0.35**	4.39±0.27**	96.22±7.11**
DM	31.26±3.25	8.14±0.26	145.86±10.86

** *p*<0.01 vs. DM group.
